# Supplementary material for: Delayed differentiation of vaginal and uterine microbiomes in dairy cows developing postpartum endometritis
Source: PLoS One. 2019 Jan 10;14(1):e0200974. doi: 10.1371/journal.pone.0200974 (PMC6328119; doi:10.1371/journal.pone.0200974)
Supplement: S2 Table — (DOCX) [file pone.0200974.s002.docx]

Supporting information - Table S2

**Delayed differentiation of vaginal and uterine microbiomes in dairy cows developing postpartum endometritis**

Raúl Miranda-CasoLuengo^1¶^*, Junnan Lu^1¶,#a^, Erin J. Williams^2¶,#b^*, Aleksandra A. Miranda-CasoLuengo^1,#c^, Stephen D. Carrington^2^, Alexander C.O. Evans^3^, Wim G. Meijer^1^

^1^ UCD School of Biomolecular and Biomedical Science and UCD Conway Institute, University College Dublin, Dublin 4, Ireland.

^2^ Veterinary Sciences Centre, UCD School of Veterinary Medicine, University College Dublin, Dublin 4, Ireland.

^3^ UCD School of Agriculture and Food Science, University College Dublin, Dublin 4, Ireland.

^#a^ Current Address: Pediatrics-Infectious Diseases, Medical School, University of Michigan, Ann Arbor, MI, USA.

^#b^ Current Address: The Roslin Institute and Royal (Dick) School of Veterinary Studies, University of Edinburgh, Easter Bush Campus, Midlothian, Scotland, EH25 9RG.

^#c^ Current Address: Moyne Institute of Preventive Medicine, Department of Microbiology, Trinity College Dublin, Dublin 2, Ireland.

*Corresponding authors

E-mail: [miranda.raul@ucd.ie](mailto:miranda.raul@ucd.ie) (RMC) and [erin.williams@ed.ac.uk](mailto:erin.williams@ed.ac.uk) (EJW)

^¶^These authors contributed equally to this work

| **Table S2. Diversity metrics of the vaginal microbiome of dairy cows at 7 days postpartum.** | | | | | | | | |
| --- | --- | --- | --- | --- | --- | --- | --- | --- |
| **Cow** | **Assignment^a^** | **Cluster^b^** | **Sequences^c^** | **OTUs^d^** | ***H'*^e^** | ***J'*^f^** | **Chao1^g^** | **Coverage^h^** |
| N697 | Healthy | I | 19913 | 2289 | 6.29 | 0.81 | 3294.69 | 88.5 |
| N356 | Healthy | I | 15305 | 1964 | 5.8 | 0.77 | 2791.9 | 87.17 |
| P77 | Healthy | I | 20025 | 985 | 5.78 | 0.84 | 1429.02 | 95.08 |
| T198 | Healthy | I | 12646 | 1234 | 5.76 | 0.81 | 1898.89 | 90.24 |
| P790 | Healthy | I | 25694 | 996 | 5.68 | 0.82 | 1261.99 | 96.12 |
| P598 | Healthy | I | 26166 | 1181 | 5.59 | 0.79 | 1669.93 | 95.49 |
| N174 | Healthy | I | 19885 | 1899 | 5.58 | 0.74 | 2784.06 | 90.45 |
| N322 | Healthy | I | 20885 | 2128 | 5.52 | 0.72 | 3105.6 | 89.81 |
| N360 | Healthy | I | 19426 | 1824 | 5.36 | 0.71 | 2657.79 | 90.61 |
| T362 | Healthy | I | 16883 | 1160 | 5.07 | 0.72 | 1625.65 | 93.13 |
| T456 | Healthy | I | 18620 | 1404 | 4.9 | 0.68 | 2365.31 | 92.46 |
| P71 | Healthy | I | 18499 | 324 | 4.71 | 0.81 | 519.56 | 98.25 |
| N678 | Healthy | I | 27194 | 687 | 4.69 | 0.72 | 977.95 | 97.47 |
| P158 | Healthy | I | 20113 | 435 | 4.66 | 0.77 | 795 | 97.84 |
| T313 | Healthy | I | 27937 | 1068 | 4.42 | 0.63 | 1567.5 | 96.18 |
| N320 | Healthy | I | 22842 | 1182 | 4.2 | 0.59 | 1681.25 | 94.83 |
| N495 | Healthy | II | 44800 | 698 | 1.32 | 0.2 | 1054.07 | 98.44 |
| T367 | Endometritis* | II | 20658 | 304 | 3.25 | 0.57 | 446.32 | 98.53 |
| P1452 | Endometritis | II | 29338 | 335 | 3.2 | 0.55 | 577 | 98.86 |
| T403 | Endometritis* | II | 14811 | 489 | 2.43 | 0.39 | 818.33 | 96.7 |
| T381 | Endometritis | II | 21421 | 382 | 1.15 | 0.19 | 854.58 | 98.22 |
| N233 | Healthy | III | 22197 | 1054 | 4.1 | 0.59 | 1491.58 | 95.25 |
| T752 | Endometritis | III | 25896 | 666 | 3.24 | 0.5 | 1341.24 | 97.43 |
| P135 | Endometritis | III | 34953 | 420 | 2.64 | 0.44 | 664.57 | 98.8 |
| P59 | Endometritis | III | 20330 | 130 | 2.2 | 0.45 | 164.13 | 99.36 |
| T462 | Endometritis | III | 20053 | 233 | 1.88 | 0.34 | 451.96 | 98.84 |
| T404 | Endometritis* | III | 33537 | 197 | 1.27 | 0.24 | 421.27 | 99.41 |
| N424 | Endometritis | III | 29601 | 348 | 0.61 | 0.1 | 756.58 | 98.82 |
| N304 | Healthy | IV | 18727 | 915 | 2.02 | 0.3 | 1222.16 | 95.11 |
| N324 | Healthy | IV | 32834 | 1056 | 1.3 | 0.19 | 1605.11 | 96.78 |
| Mean (SD) | Overall |  | 23373 (6897.7) | 932.9 (614.2) | 3.82 (1.75) | 0.57 (0.23) | 1409.8 (860) | 95.5  (3.6) |
|  | Healthy |  | 22529.6 (7043.6) | 1224.2 (544.8) | 4.64 (1.46) | 0.66 (0.2) | 1790 (789.6) | 93.9  (3.4) |
|  | Endometritis |  | 25059.8 (6620.3) | 350.4 (153.8) | 2.19 (0.94) | 0.38 (0.16) | 649.6 (322.1) | 98.5  (0.9) |
| P^i^ |  |  | 0.3462 | < 0.001 | < 0.001 | < 0.001 | < 0.001 | < 0.001 |
| ^a^ Clinical assignments were made retrospectively from the assessment of vaginal mucus as defined by Sheldon et. al. (2006). Vaginal mucus scores (VMS) of 3 at 21 DPP or ≥2 after 26 DPP were used as cut-offs for the assignment of endometritis. * These assignments did not meet the Sheldon definition but were considered borderline given that they produced a VMS of 3 at 7 and 14 DPP and VMS of 2 at 21 DPP, meeting the Williams et. al. (2005) criteria. Note that the vaginal microbiome of these three cows were distinct from healthy cows and clustered together with endometritic cows that did meet Sheldon's definition.  ^b^ Cluster membership defined by Weighted Unifrac Hierarchical Clustering (Figure 6A). See Table S2 for a summary of metrics per cluster.  ^c^ Number of sequences per sample after trimming, quality control and removal of chimeras.  ^d^ Operational Taxonomic Units at 97% of identity.  ^e^ Shannon index of diversity.  ^f^ Species Evenness.  ^g^ Estimation of total species richness.  ^h^ Estimation of the percent of the total species represented in each sample.  ^i^ P values are based on T-test | | | | | | | | |

**References**

Sheldon IM, Lewis GS, LeBlanc S, Gilbert RO. Defining postpartum uterine disease in cattle. Theriogenology. 2006; 65: 1516-1530.

Williams EJ, Fischer DP, Pfeiffer DU, England GC, Noakes DE, Dobson H, Sheldon IM. Clinical evaluation of postpartum vaginal mucus reflects uterine bacterial infection and the immune response in cattle. Theriogenology. 2005; 63: 102-117.
