# Supplementary material for: Delayed differentiation of vaginal and uterine microbiomes in dairy cows developing postpartum endometritis
Source: PLoS One. 2019 Jan 10;14(1):e0200974. doi: 10.1371/journal.pone.0200974 (PMC6328119; doi:10.1371/journal.pone.0200974)
Supplement: S1 Fig — The relative abundance of experimentally determined terminal restriction fragments (TRF) was calculated as the average fluorescence signal associated with the TRF in the vagina of sampled animals. In silico T-RFLP profiles were generated using the dataset of quality-controlled sequences obtained by pyrosequencing from vaginal microbiomes of cows at 7 DPP. The position of the first MspI restriction site was determined for each sequence. Given that the primers had previously been removed during the bioinformatic pipeline, the length of the 27f primer (20 bp) was added for the calculation of the length of TRFs. The relative abundance of each in silico TRF was subsequently determined from their frequency relative to the total number of sequences in the dataset. The relative abundances of the two TRFs exceeding the scale are 23.7% and 10.1% from left to right. This comparison does not attempt to match each experimental TRF to a given sequence or taxonomic affiliation. (DOCX) [file pone.0200974.s003.docx]

Supporting information - Figure S1

**Delayed differentiation of vaginal and uterine microbiomes in dairy cows developing postpartum endometritis**

Raúl Miranda-CasoLuengo^1¶^*, Junnan Lu^1¶,#a^, Erin J. Williams^2¶,#b^*, Aleksandra A. Miranda-CasoLuengo^1,#c^, Stephen D. Carrington^2^, Alexander C.O. Evans^3^, Wim G. Meijer^1^

^1^ UCD School of Biomolecular and Biomedical Science and UCD Conway Institute, University College Dublin, Dublin 4, Ireland.

^2^ Veterinary Sciences Centre, UCD School of Veterinary Medicine, University College Dublin, Dublin 4, Ireland.

^3^ UCD School of Agriculture and Food Science, University College Dublin, Dublin 4, Ireland.

^#a^ Current Address: Pediatrics-Infectious Diseases, Medical School, University of Michigan, Ann Arbor, MI, USA.

^#b^ Current Address: The Roslin Institute and Royal (Dick) School of Veterinary Studies, University of Edinburgh, Easter Bush Campus, Midlothian, Scotland, EH25 9RG.

^#c^ Current Address: Moyne Institute of Preventive Medicine, Department of Microbiology, Trinity College Dublin, Dublin 2, Ireland.

*Corresponding authors

E-mail: [miranda.raul@ucd.ie](mailto:miranda.raul@ucd.ie) (RMC) and [erin.williams@ed.ac.uk](mailto:erin.williams@ed.ac.uk) (EJW)

^¶^These authors contributed equally to this work


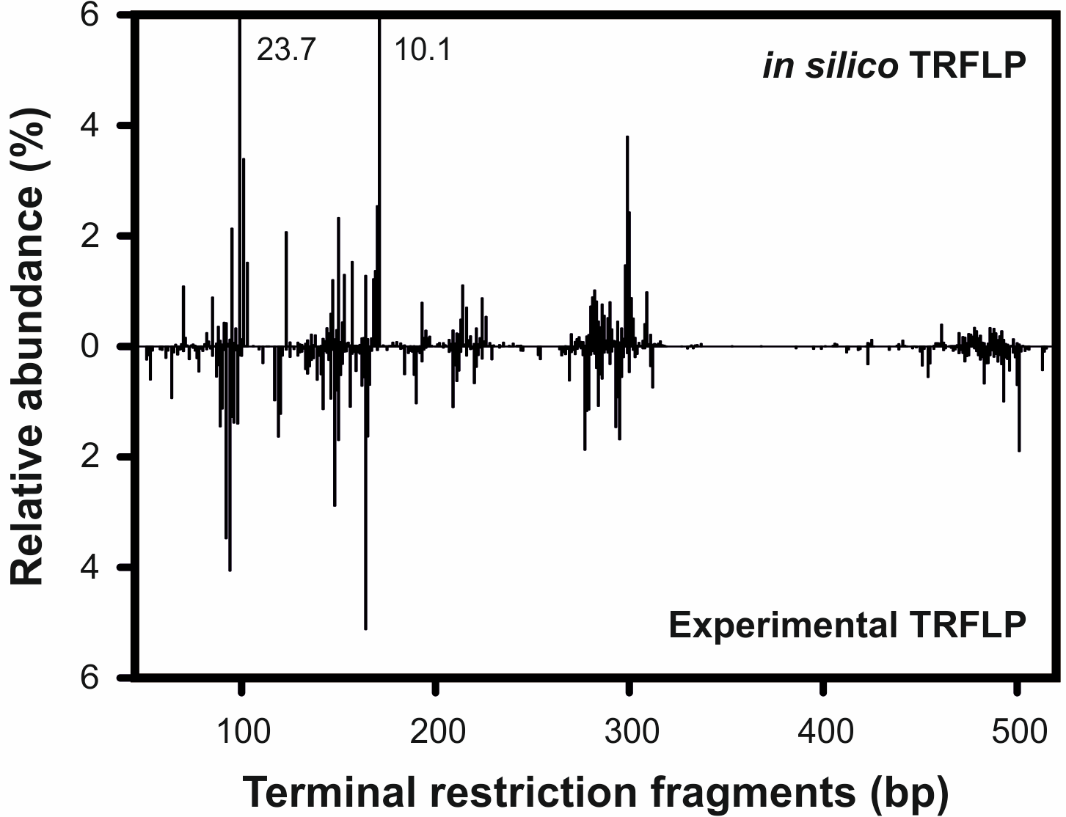


**Figure S1. Comparison of experimental and *in silico* generated TRFLP profiles.** The relative abundance of experimentally determined terminal restriction fragments (TRF) was calculated as the average fluorescence signal associated with the TRF in the vagina of sampled animals. *In silico* TRFLP profiles were generated using the dataset of quality-controlled sequences obtained by pyrosequencing from vaginal microbiomes of cows at 7 DPP. The position of the first *Msp*I restriction site was determined for each sequence. Given that the primers had previously been removed during the bioinformatic pipeline, the length of the 27f primer (20 bp) was added for the calculation of the length of TRFs. The relative abundance of each *in silico* TRF was subsequently determined from the frequency of each TRF relative to the total number of sequences in the dataset. The relative abundances of the two TRFs exceeding the scale are 23.7% and 10.1% from left to right. This comparison does not attempt to match each experimental TRF to a given sequence or taxonomic affiliation.
