# Supplementary material for: Delayed differentiation of vaginal and uterine microbiomes in dairy cows developing postpartum endometritis
Source: PLoS One. 2019 Jan 10;14(1):e0200974. doi: 10.1371/journal.pone.0200974 (PMC6328119; doi:10.1371/journal.pone.0200974)
Supplement: S2 Fig — The microbiomes associated with vaginal samples obtained from cows before calving were compared in a resemblance matrix based on the Bray-Curtis similarity. The health status of each cow was assessed depending on the outcome of the transient postpartum infection. The tips of the branches are colour coded according to the outcome of postpartum health status: Black, non-susceptible; Red, susceptible to postpartum endometritis. Community profiles were determined by T-RFLP of the 16S rRNA as described in the section of Materials and Methods. Analysis was performed in PRIMER6 and the figure was re-drawn in CorelDraw X4. (DOCX) [file pone.0200974.s004.docx]

Supporting information - Figure S2

**Delayed differentiation of vaginal and uterine microbiomes in dairy cows developing postpartum endometritis**

Raúl Miranda-CasoLuengo^1¶^*, Junnan Lu^1¶,#a^, Erin J. Williams^2¶,#b^*, Aleksandra A. Miranda-CasoLuengo^1,#c^, Stephen D. Carrington^2^, Alexander C.O. Evans^3^, Wim G. Meijer^1^

^1^ UCD School of Biomolecular and Biomedical Science and UCD Conway Institute, University College Dublin, Dublin 4, Ireland.

^2^ Veterinary Sciences Centre, UCD School of Veterinary Medicine, University College Dublin, Dublin 4, Ireland.

^3^ UCD School of Agriculture and Food Science, University College Dublin, Dublin 4, Ireland.

^#a^ Current Address: Pediatrics-Infectious Diseases, Medical School, University of Michigan, Ann Arbor, MI, USA.

^#b^ Current Address: The Roslin Institute and Royal (Dick) School of Veterinary Studies, University of Edinburgh, Easter Bush Campus, Midlothian, Scotland, EH25 9RG.

^#c^ Current Address: Moyne Institute of Preventive Medicine, Department of Microbiology, Trinity College Dublin, Dublin 2, Ireland.

*Corresponding authors

E-mail: [miranda.raul@ucd.ie](mailto:miranda.raul@ucd.ie) (RMC) and [erin.williams@ed.ac.uk](mailto:erin.williams@ed.ac.uk) (EJW)

^¶^These authors contributed equally to this work


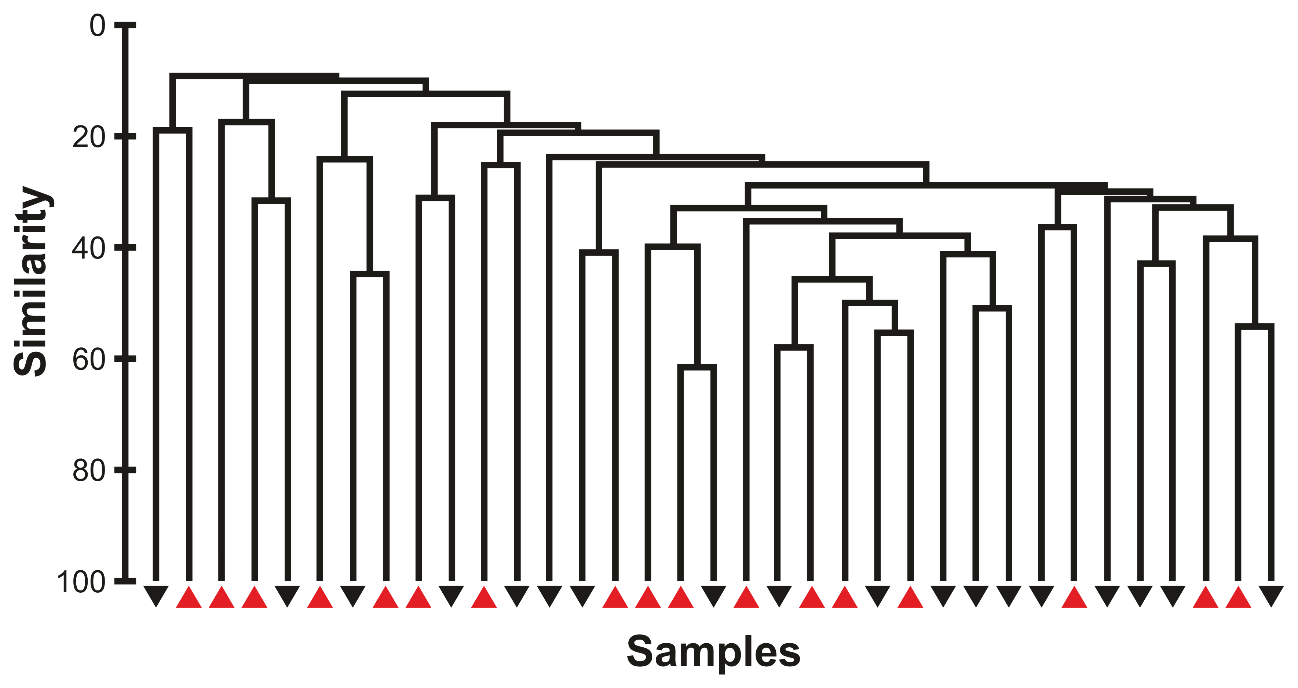


**Figure S2. Hierarchical cluster analysis of pre-calving dairy cows based of their vaginal microbiomes.** The microbiomes associated with vaginal samples obtained from cows before calving were compared in a resemblance matrix based on the Bray-Curtis similarity. The health status of each cow was assessed depending on the outcome of the transient postpartum infection. The tips of the branches are colour coded according to the outcome of postpartum health status: Black, non-susceptible; Red, susceptible to postpartum endometritis. Community profiles were determined by T-RFLP of the 16S rRNA as described in the section of Materials and Methods. Analysis was performed in PRIMER6 and the figure was re-drawn in CorelDraw X4.
