# Supplementary material for: Delayed differentiation of vaginal and uterine microbiomes in dairy cows developing postpartum endometritis
Source: PLoS One. 2019 Jan 10;14(1):e0200974. doi: 10.1371/journal.pone.0200974 (PMC6328119; doi:10.1371/journal.pone.0200974)
Supplement: S3 Fig — Taxonomic composition at phylum and genus levels, respectively. Each bar represents the average of the vaginal microbiome in each of the following categories: A) Clinical assignment. h, healthy; e, endometritis B) Cluster as defined in Fig 6A, C) Farm of collection. While there is a farm effect in the taxonomic composition of the vaginal microbiome at genus level, its contribution is lower than the effect observed by health status. The colour key of selected phyla (within box) and genera from A, B and C is placed at the right of the figure. Superscripts: a OTUs with ambiguous assignment below the indicated taxonomic level; b OTUs that although matching reference sequences in the Greengenes 13_8 database, no taxonomic name has been defined. In these cases, the lowest taxonomic name is provided; c OTUs matching reference sequences for which taxonomic changes above the rank of genus have been recommend by Greengenes based on whole genome phylogeny; d OTUs matching Genus name contested. (DOCX) [file pone.0200974.s005.docx]

Supporting information - Figure S3

**Delayed differentiation of vaginal and uterine microbiomes in dairy cows developing postpartum endometritis**

Raúl Miranda-CasoLuengo^1¶^*, Junnan Lu^1¶,#a^, Erin J. Williams^2¶,#b^*, Aleksandra A. Miranda-CasoLuengo^1,#c^, Stephen D. Carrington^2^, Alexander C.O. Evans^3^, Wim G. Meijer^1^

^1^ UCD School of Biomolecular and Biomedical Science and UCD Conway Institute, University College Dublin, Dublin 4, Ireland.

^2^ Veterinary Sciences Centre, UCD School of Veterinary Medicine, University College Dublin, Dublin 4, Ireland.

^3^ UCD School of Agriculture and Food Science, University College Dublin, Dublin 4, Ireland.

^#a^ Current Address: Pediatrics-Infectious Diseases, Medical School, University of Michigan, Ann Arbor, MI, USA.

^#b^ Current Address: The Roslin Institute and Royal (Dick) School of Veterinary Studies, University of Edinburgh, Easter Bush Campus, Midlothian, Scotland, EH25 9RG.

^#c^ Current Address: Moyne Institute of Preventive Medicine, Department of Microbiology, Trinity College Dublin, Dublin 2, Ireland.

*Corresponding authors

E-mail: [miranda.raul@ucd.ie](mailto:miranda.raul@ucd.ie) (RMC) and [erin.williams@ed.ac.uk](mailto:erin.williams@ed.ac.uk) (EJW)

^¶^These authors contributed equally to this work


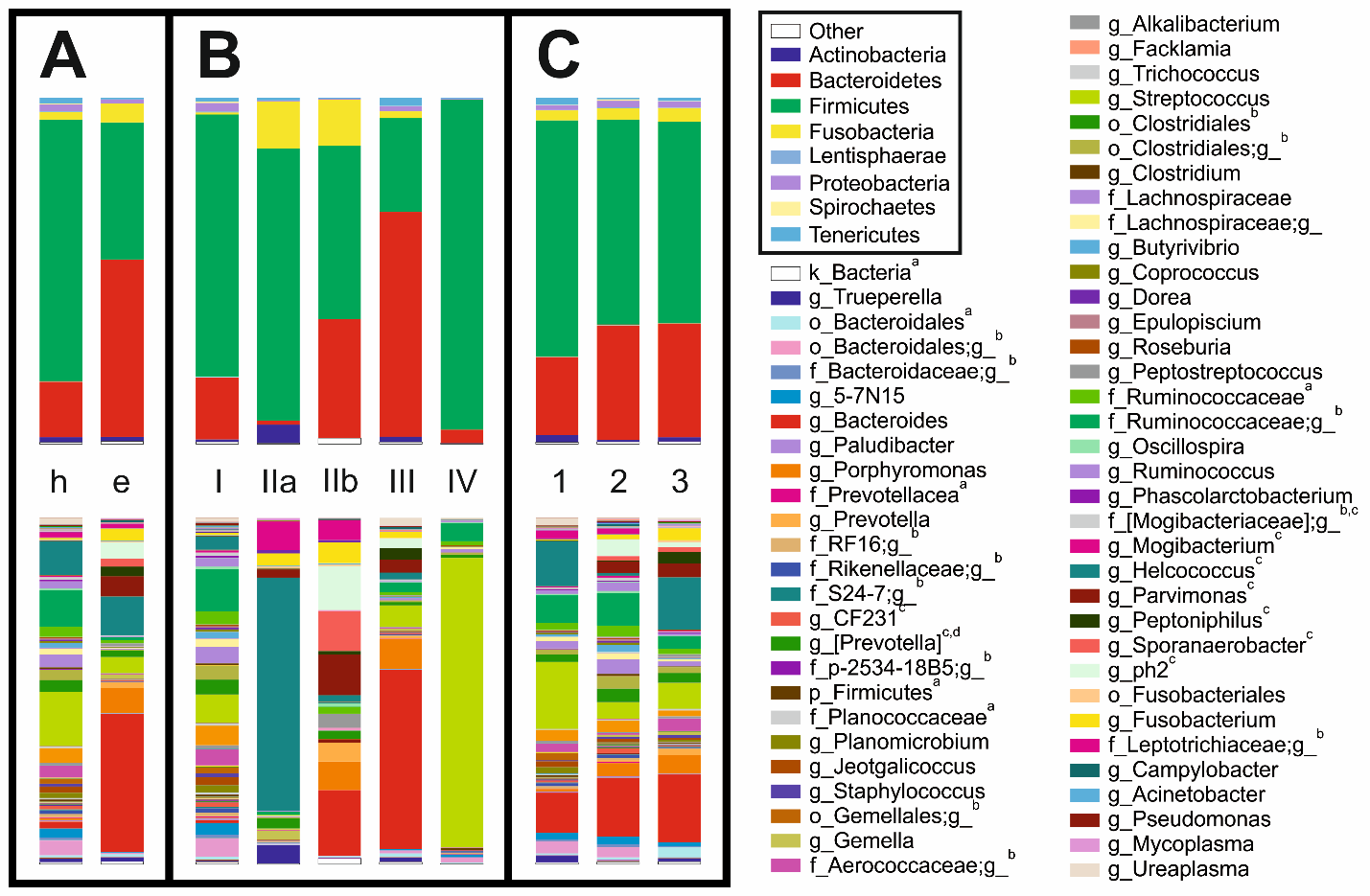


**Figure S3. Category-based taxonomic composition of the vaginal microbiome of cows at 7 DPP.** Taxonomic composition at phylum and genus levels, respectively. Each bar represents the average of the vaginal microbiome in each of the following categories: **A)** Clinical assignment. h, healthy; e, endometritis **B)** Cluster as defined in Figure 6A, **C)** Farm of collection. While there is a farm effect in the taxonomic composition of the vaginal microbiome at genus level, its contribution is lower than the effect observed by health status. The colour key of selected phyla (within box) and genera from A, B and C is placed at the right of the figure. Superscripts: **^a^** OTUs with ambiguous assignment below the indicated taxonomic level; **^b^** OTUs that although matching reference sequences in the Greengenes 13_8 database, no taxonomic name has been defined. In these cases, the lowest taxonomic name is provided; **^c^** OTUs matching reference sequences for which taxonomic changes above the rank of genus have been recommend by Greengenes based on whole genome phylogeny; **^d^** OTUs matching Genus name contested.
