# Supplementary material for: Delayed differentiation of vaginal and uterine microbiomes in dairy cows developing postpartum endometritis
Source: PLoS One. 2019 Jan 10;14(1):e0200974. doi: 10.1371/journal.pone.0200974 (PMC6328119; doi:10.1371/journal.pone.0200974)
Supplement: S4 Fig — Original output generated by QIIME. To visualise it double click on bar_charts.html. (ZIP) [file pone.0200974.s006.zip › Figure S4/charts/eBS9C7dWWZM30zwPdT8QOjW40KfYD9_legend.pdf]

Unclassified;Other;Other
k__Bacteria;Other;Other
k__Bacteria;p__Acidobacteria;Other
k__Bacteria;p__Acidobacteria;c__Acidobacteria-6
k__Bacteria;p__Acidobacteria;c__Acidobacteriia
k__Bacteria;p__Acidobacteria;c__[Chloracidobacteria]
k__Bacteria;p__Acidobacteria;c__iii1-8
k__Bacteria;p__Actinobacteria;Other
k__Bacteria;p__Actinobacteria;c__Acidimicrobiia
k__Bacteria;p__Actinobacteria;c__Actinobacteria
k__Bacteria;p__Actinobacteria;c__Coriobacteriia
k__Bacteria;p__Actinobacteria;c__OPB41
k__Bacteria;p__Actinobacteria;c__Thermoleophilia
k__Bacteria;p__Bacteroidetes;Other
k__Bacteria;p__Bacteroidetes;c__Bacteroidia
k__Bacteria;p__Bacteroidetes;c__Cytophagia
k__Bacteria;p__Bacteroidetes;c__Flavobacteriia
k__Bacteria;p__Bacteroidetes;c__Sphingobacteriia
k__Bacteria;p__Bacteroidetes;c__[Rhodothermi]
k__Bacteria;p__Bacteroidetes;c__[Saprospirae]
k__Bacteria;p__Chloroflexi;c__Anaerolineae
k__Bacteria;p__Chloroflexi;c__Thermomicrobia
k__Bacteria;p__Cyanobacteria;Other
k__Bacteria;p__Cyanobacteria;c__4C0d-2
k__Bacteria;p__Cyanobacteria;c__Chloroplast
k__Bacteria;p__Elusimicrobia;c__Elusimicrobia
k__Bacteria;p__FBP;c__
k__Bacteria;p__Fibrobacteres;c__Fibrobacteria
k__Bacteria;p__Firmicutes;Other
k__Bacteria;p__Firmicutes;c__Bacilli
k__Bacteria;p__Firmicutes;c__Clostridia
k__Bacteria;p__Firmicutes;c__Erysipelotrichi
k__Bacteria;p__Fusobacteria;c__Fusobacteriia
k__Bacteria;p__Gemmatimonadetes;c__Gemmatimonadetes
k__Bacteria;p__Lentisphaerae;c__[Lentisphaeria]
k__Bacteria;p__OD1;Other
k__Bacteria;p__Planctomycetes;c__Planctomycetia
k__Bacteria;p__Proteobacteria;Other
k__Bacteria;p__Proteobacteria;c__Alphaproteobacteria
k__Bacteria;p__Proteobacteria;c__Betaproteobacteria
k__Bacteria;p__Proteobacteria;c__Deltaproteobacteria
k__Bacteria;p__Proteobacteria;c__Epsilonproteobacteria
k__Bacteria;p__Proteobacteria;c__Gammaproteobacteria
k__Bacteria;p__Spirochaetes;c__Spirochaetes
k__Bacteria;p__Synergistetes;c__Synergistia
k__Bacteria;p__TM7;c__TM7-1
k__Bacteria;p__TM7;c__TM7-3
k__Bacteria;p__Tenericutes;Other
k__Bacteria;p__Tenericutes;c__Mollicutes
k__Bacteria;p__Tenericutes;c__RF3
k__Bacteria;p__Verrucomicrobia;c__Opitutae
k__Bacteria;p__Verrucomicrobia;c__Verruco-5
k__Bacteria;p__Verrucomicrobia;c__Verrucomicrobiae
k__Bacteria;p__[Thermi];c__Deinococci
