# Supplementary material for: Delayed differentiation of vaginal and uterine microbiomes in dairy cows developing postpartum endometritis
Source: PLoS One. 2019 Jan 10;14(1):e0200974. doi: 10.1371/journal.pone.0200974 (PMC6328119; doi:10.1371/journal.pone.0200974)
Supplement: S4 Fig — Original output generated by QIIME. To visualise it double click on bar_charts.html. (ZIP) [file pone.0200974.s006.zip › Figure S4/charts/MtCoXE44sIKHPIy1aZDYncrCSc2azG_legend.pdf]

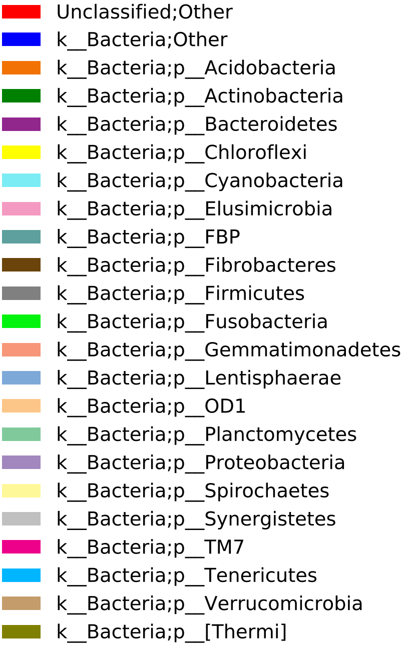
Unclassified;Other
k__Bacteria;Other
k__Bacteria;p__Acidobacteria
k__Bacteria;p__Actinobacteria
k__Bacteria;p__Bacteroidetes
k__Bacteria;p__Chloroflexi
k__Bacteria;p__Cyanobacteria
k__Bacteria;p__Elusimicrobia
k__Bacteria;p__FBP
k__Bacteria;p__Fibrobacteres
k__Bacteria;p__Firmicutes
k__Bacteria;p__Fusobacteria
k__Bacteria;p__Gemmatimonadetes
k__Bacteria;p__Lentisphaerae
k__Bacteria;p__OD1
k__Bacteria;p__Planctomycetes
k__Bacteria;p__Proteobacteria
k__Bacteria;p__Spirochaetes
k__Bacteria;p__Synergistetes
k__Bacteria;p__TM7
k__Bacteria;p__Tenericutes
k__Bacteria;p__Verrucomicrobia
k__Bacteria;p__[Thermi]
