# Supplementary material for: Delayed differentiation of vaginal and uterine microbiomes in dairy cows developing postpartum endometritis
Source: PLoS One. 2019 Jan 10;14(1):e0200974. doi: 10.1371/journal.pone.0200974 (PMC6328119; doi:10.1371/journal.pone.0200974)
Supplement: S4 Fig — Original output generated by QIIME. To visualise it double click on bar_charts.html. (ZIP) [file pone.0200974.s006.zip › Figure S4/charts/sZ7JijCfSoGszo0E0RuyzXLmljCn0t_legend.pdf]

Unclassified;Other;Other;Other
k__Bacteria;Other;Other;Other
k__Bacteria;p__Acidobacteria;Other;Other
k__Bacteria;p__Acidobacteria;c__Acidobacteria-6;Other
k__Bacteria;p__Acidobacteria;c__Acidobacteria-6;o__iii1-15
k__Bacteria;p__Acidobacteria;c__Acidobacteriia;o__Acidobacteriales
k__Bacteria;p__Acidobacteria;c__[Chloracidobacteria];o__RB41
k__Bacteria;p__Acidobacteria;c__iii1-8;o__DS-18
k__Bacteria;p__Actinobacteria;Other;Other
k__Bacteria;p__Actinobacteria;c__Acidimicrobiia;o__Acidimicrobiales
k__Bacteria;p__Actinobacteria;c__Actinobacteria;o__Actinomycetales
k__Bacteria;p__Actinobacteria;c__Actinobacteria;o__Bifidobacteriales
k__Bacteria;p__Actinobacteria;c__Coriobacteriia;o__Coriobacteriales
k__Bacteria;p__Actinobacteria;c__OPB41;o__
k__Bacteria;p__Actinobacteria;c__Thermoleophilia;o__Solirubrobacterales
k__Bacteria;p__Bacteroidetes;Other;Other
k__Bacteria;p__Bacteroidetes;c__Bacteroidia;o__Bacteroidales
k__Bacteria;p__Bacteroidetes;c__Cytophagia;o__Cytophagales
k__Bacteria;p__Bacteroidetes;c__Flavobacteriia;o__Flavobacteriales
k__Bacteria;p__Bacteroidetes;c__Sphingobacteriia;o__Sphingobacteriales
k__Bacteria;p__Bacteroidetes;c__[Rhodothermi];o__[Rhodothermales]
k__Bacteria;p__Bacteroidetes;c__[Saprospirae];o__[Saprospirales]
k__Bacteria;p__Chloroflexi;c__Anaerolineae;o__Anaerolineales
k__Bacteria;p__Chloroflexi;c__Thermomicrobia;o__JG30-KF-CM45
k__Bacteria;p__Cyanobacteria;Other;Other
k__Bacteria;p__Cyanobacteria;c__4C0d-2;o__MLE1-12
k__Bacteria;p__Cyanobacteria;c__4C0d-2;o__YS2
k__Bacteria;p__Cyanobacteria;c__Chloroplast;o__Streptophyta
k__Bacteria;p__Elusimicrobia;c__Elusimicrobia;o__Elusimicrobiales
k__Bacteria;p__FBP;c__;o__
k__Bacteria;p__Fibrobacteres;c__Fibrobacteria;o__Fibrobacterales
k__Bacteria;p__Firmicutes;Other;Other
k__Bacteria;p__Firmicutes;c__Bacilli;Other
k__Bacteria;p__Firmicutes;c__Bacilli;o__Bacillales
k__Bacteria;p__Firmicutes;c__Bacilli;o__Gemellales
k__Bacteria;p__Firmicutes;c__Bacilli;o__Lactobacillales
k__Bacteria;p__Firmicutes;c__Bacilli;o__Turicibacterales
k__Bacteria;p__Firmicutes;c__Clostridia;Other
k__Bacteria;p__Firmicutes;c__Clostridia;o__Clostridiales
k__Bacteria;p__Firmicutes;c__Erysipelotrichi;o__Erysipelotrichales
k__Bacteria;p__Fusobacteria;c__Fusobacteriia;o__Fusobacteriales
k__Bacteria;p__Gemmatimonadetes;c__Gemmatimonadetes;o__
k__Bacteria;p__Lentisphaerae;c__[Lentisphaeria];o__Victivallales
k__Bacteria;p__Lentisphaerae;c__[Lentisphaeria];o__Z20
k__Bacteria;p__OD1;Other;Other
k__Bacteria;p__Planctomycetes;c__Planctomycetia;Other
k__Bacteria;p__Planctomycetes;c__Planctomycetia;o__Pirellulales
k__Bacteria;p__Proteobacteria;Other;Other
k__Bacteria;p__Proteobacteria;c__Alphaproteobacteria;Other
k__Bacteria;p__Proteobacteria;c__Alphaproteobacteria;o__Caulobacterales
k__Bacteria;p__Proteobacteria;c__Alphaproteobacteria;o__RF32
k__Bacteria;p__Proteobacteria;c__Alphaproteobacteria;o__Rhizobiales
k__Bacteria;p__Proteobacteria;c__Alphaproteobacteria;o__Rhodobacterales
k__Bacteria;p__Proteobacteria;c__Alphaproteobacteria;o__Rhodospirillales
k__Bacteria;p__Proteobacteria;c__Alphaproteobacteria;o__Rickettsiales
k__Bacteria;p__Proteobacteria;c__Alphaproteobacteria;o__Sphingomonadales
k__Bacteria;p__Proteobacteria;c__Betaproteobacteria;Other
k__Bacteria;p__Proteobacteria;c__Betaproteobacteria;o__ASSO-13
k__Bacteria;p__Proteobacteria;c__Betaproteobacteria;o__Burkholderiales
k__Bacteria;p__Proteobacteria;c__Betaproteobacteria;o__Neisseriales
k__Bacteria;p__Proteobacteria;c__Betaproteobacteria;o__SC-I-84
k__Bacteria;p__Proteobacteria;c__Deltaproteobacteria;Other
k__Bacteria;p__Proteobacteria;c__Deltaproteobacteria;o__Desulfovibrionales
k__Bacteria;p__Proteobacteria;c__Epsilonproteobacteria;o__Campylobacterales
k__Bacteria;p__Proteobacteria;c__Gammaproteobacteria;Other
k__Bacteria;p__Proteobacteria;c__Gammaproteobacteria;o__Aeromonadales
k__Bacteria;p__Proteobacteria;c__Gammaproteobacteria;o__Alteromonadales
k__Bacteria;p__Proteobacteria;c__Gammaproteobacteria;o__Enterobacteriales
k__Bacteria;p__Proteobacteria;c__Gammaproteobacteria;o__Legionellales
k__Bacteria;p__Proteobacteria;c__Gammaproteobacteria;o__Oceanospirillales
k__Bacteria;p__Proteobacteria;c__Gammaproteobacteria;o__Pasteurellales
k__Bacteria;p__Proteobacteria;c__Gammaproteobacteria;o__Pseudomonadales
k__Bacteria;p__Proteobacteria;c__Gammaproteobacteria;o__Xanthomonadales
k__Bacteria;p__Spirochaetes;c__Spirochaetes;o__M2PT2-76
k__Bacteria;p__Spirochaetes;c__Spirochaetes;o__Sphaerochaetales
k__Bacteria;p__Spirochaetes;c__Spirochaetes;o__Spirochaetales
k__Bacteria;p__Synergistetes;c__Synergistia;o__Synergistales
k__Bacteria;p__TM7;c__TM7-1;o__
k__Bacteria;p__TM7;c__TM7-3;Other
k__Bacteria;p__TM7;c__TM7-3;o__CW040
k__Bacteria;p__TM7;c__TM7-3;o__EW055
k__Bacteria;p__Tenericutes;Other;Other
k__Bacteria;p__Tenericutes;c__Mollicutes;o__Acholeplasmatales
k__Bacteria;p__Tenericutes;c__Mollicutes;o__Anaeroplasmatales
k__Bacteria;p__Tenericutes;c__Mollicutes;o__Mycoplasmatales
k__Bacteria;p__Tenericutes;c__Mollicutes;o__RF39
k__Bacteria;p__Tenericutes;c__RF3;o__ML615J-28
k__Bacteria;p__Verrucomicrobia;c__Opitutae;o__HA64
k__Bacteria;p__Verrucomicrobia;c__Opitutae;o__[Cerasicoccales]
k__Bacteria;p__Verrucomicrobia;c__Verruco-5;o__WCHB1-41
k__Bacteria;p__Verrucomicrobia;c__Verrucomicrobiae;o__Verrucomicrobiales
k__Bacteria;p__[Thermi];c__Deinococci;o__Deinococcales
