# Supplementary material for: Delayed differentiation of vaginal and uterine microbiomes in dairy cows developing postpartum endometritis
Source: PLoS One. 2019 Jan 10;14(1):e0200974. doi: 10.1371/journal.pone.0200974 (PMC6328119; doi:10.1371/journal.pone.0200974)
Supplement: S4 Fig — Original output generated by QIIME. To visualise it double click on bar_charts.html. (ZIP) [file pone.0200974.s006.zip › Figure S4/charts/TXgz38RRizZhyyZ1fpeSyCdk3gq0jj_legend.pdf]

Unclassified;Other;Other;Other;Other
k__Bacteria;Other;Other;Other;Other
k__Bacteria;p__Acidobacteria;Other;Other;Other
k__Bacteria;p__Acidobacteria;c__Acidobacteria-6;Other;Other
k__Bacteria;p__Acidobacteria;c__Acidobacteria-6;o__iii1-15;f__
k__Bacteria;p__Acidobacteria;c__Acidobacteriia;o__Acidobacteriales;f__Acidobacteriaceae
k__Bacteria;p__Acidobacteria;c__[Chloracidobacteria];o__RB41;f__Ellin6075
k__Bacteria;p__Acidobacteria;c__iii1-8;o__DS-18;f__
k__Bacteria;p__Actinobacteria;Other;Other;Other
k__Bacteria;p__Actinobacteria;c__Acidimicrobiia;o__Acidimicrobiales;f__
k__Bacteria;p__Actinobacteria;c__Acidimicrobiia;o__Acidimicrobiales;f__C111
k__Bacteria;p__Actinobacteria;c__Acidimicrobiia;o__Acidimicrobiales;f__Microthrixaceae
k__Bacteria;p__Actinobacteria;c__Actinobacteria;o__Actinomycetales;Other
k__Bacteria;p__Actinobacteria;c__Actinobacteria;o__Actinomycetales;f__
k__Bacteria;p__Actinobacteria;c__Actinobacteria;o__Actinomycetales;f__Actinomycetaceae
k__Bacteria;p__Actinobacteria;c__Actinobacteria;o__Actinomycetales;f__Brevibacteriaceae
k__Bacteria;p__Actinobacteria;c__Actinobacteria;o__Actinomycetales;f__Cellulomonadaceae
k__Bacteria;p__Actinobacteria;c__Actinobacteria;o__Actinomycetales;f__Corynebacteriaceae
k__Bacteria;p__Actinobacteria;c__Actinobacteria;o__Actinomycetales;f__Dermabacteraceae
k__Bacteria;p__Actinobacteria;c__Actinobacteria;o__Actinomycetales;f__Dietziaceae
k__Bacteria;p__Actinobacteria;c__Actinobacteria;o__Actinomycetales;f__Intrasporangiaceae
k__Bacteria;p__Actinobacteria;c__Actinobacteria;o__Actinomycetales;f__Microbacteriaceae
k__Bacteria;p__Actinobacteria;c__Actinobacteria;o__Actinomycetales;f__Micrococcaceae
k__Bacteria;p__Actinobacteria;c__Actinobacteria;o__Actinomycetales;f__Nocardiaceae
k__Bacteria;p__Actinobacteria;c__Actinobacteria;o__Actinomycetales;f__Nocardioidaceae
k__Bacteria;p__Actinobacteria;c__Actinobacteria;o__Actinomycetales;f__Propionibacteriaceae
k__Bacteria;p__Actinobacteria;c__Actinobacteria;o__Actinomycetales;f__Sanguibacteraceae
k__Bacteria;p__Actinobacteria;c__Actinobacteria;o__Actinomycetales;f__Yaniellaceae
k__Bacteria;p__Actinobacteria;c__Actinobacteria;o__Bifidobacteriales;f__Bifidobacteriaceae
k__Bacteria;p__Actinobacteria;c__Coriobacteriia;o__Coriobacteriales;f__Coriobacteriaceae
k__Bacteria;p__Actinobacteria;c__OPB41;o__;f__
k__Bacteria;p__Actinobacteria;c__Thermoleophilia;o__Solirubrobacterales;Other
k__Bacteria;p__Actinobacteria;c__Thermoleophilia;o__Solirubrobacterales;f__Conexibacteraceae
k__Bacteria;p__Actinobacteria;c__Thermoleophilia;o__Solirubrobacterales;f__Patulibacteraceae
k__Bacteria;p__Bacteroidetes;Other;Other;Other
k__Bacteria;p__Bacteroidetes;c__Bacteroidia;o__Bacteroidales;Other
k__Bacteria;p__Bacteroidetes;c__Bacteroidia;o__Bacteroidales;f__
k__Bacteria;p__Bacteroidetes;c__Bacteroidia;o__Bacteroidales;f__BS11
k__Bacteria;p__Bacteroidetes;c__Bacteroidia;o__Bacteroidales;f__Bacteroidaceae
k__Bacteria;p__Bacteroidetes;c__Bacteroidia;o__Bacteroidales;f__Marinilabiaceae
k__Bacteria;p__Bacteroidetes;c__Bacteroidia;o__Bacteroidales;f__Porphyromonadaceae
k__Bacteria;p__Bacteroidetes;c__Bacteroidia;o__Bacteroidales;f__Prevotellaceae
k__Bacteria;p__Bacteroidetes;c__Bacteroidia;o__Bacteroidales;f__RF16
k__Bacteria;p__Bacteroidetes;c__Bacteroidia;o__Bacteroidales;f__Rikenellaceae
k__Bacteria;p__Bacteroidetes;c__Bacteroidia;o__Bacteroidales;f__S24-7
k__Bacteria;p__Bacteroidetes;c__Bacteroidia;o__Bacteroidales;f__[Barnesiellaceae]
k__Bacteria;p__Bacteroidetes;c__Bacteroidia;o__Bacteroidales;f__[Odoribacteraceae]
k__Bacteria;p__Bacteroidetes;c__Bacteroidia;o__Bacteroidales;f__[Paraprevotellaceae]
k__Bacteria;p__Bacteroidetes;c__Bacteroidia;o__Bacteroidales;f__p-2534-18B5
k__Bacteria;p__Bacteroidetes;c__Cytophagia;o__Cytophagales;f__Cyclobacteriaceae
k__Bacteria;p__Bacteroidetes;c__Cytophagia;o__Cytophagales;f__Cytophagaceae
k__Bacteria;p__Bacteroidetes;c__Flavobacteriia;o__Flavobacteriales;f__Cryomorphaceae
k__Bacteria;p__Bacteroidetes;c__Flavobacteriia;o__Flavobacteriales;f__Flavobacteriaceae
k__Bacteria;p__Bacteroidetes;c__Flavobacteriia;o__Flavobacteriales;f__[Weeksellaceae]
k__Bacteria;p__Bacteroidetes;c__Sphingobacteriia;o__Sphingobacteriales;f__Sphingobacteriaceae
k__Bacteria;p__Bacteroidetes;c__[Rhodothermi];o__[Rhodothermales];f__[Balneolaceae]
k__Bacteria;p__Bacteroidetes;c__[Saprospirae];o__[Saprospirales];f__Chitinophagaceae
k__Bacteria;p__Chloroflexi;c__Anaerolineae;o__Anaerolineales;f__Anaerolinaceae
k__Bacteria;p__Chloroflexi;c__Thermomicrobia;o__JG30-KF-CM45;f__
k__Bacteria;p__Cyanobacteria;Other;Other;Other
k__Bacteria;p__Cyanobacteria;c__4C0d-2;o__MLE1-12;f__
k__Bacteria;p__Cyanobacteria;c__4C0d-2;o__YS2;f__
k__Bacteria;p__Cyanobacteria;c__Chloroplast;o__Streptophyta;f__
k__Bacteria;p__Elusimicrobia;c__Elusimicrobia;o__Elusimicrobiales;f__Elusimicrobiaceae
k__Bacteria;p__FBP;c__;o__;f__
k__Bacteria;p__Fibrobacteres;c__Fibrobacteria;o__Fibrobacterales;f__Fibrobacteraceae
k__Bacteria;p__Firmicutes;Other;Other;Other
k__Bacteria;p__Firmicutes;c__Bacilli;Other;Other
k__Bacteria;p__Firmicutes;c__Bacilli;o__Bacillales;Other
k__Bacteria;p__Firmicutes;c__Bacilli;o__Bacillales;f__Bacillaceae
k__Bacteria;p__Firmicutes;c__Bacilli;o__Bacillales;f__Listeriaceae
k__Bacteria;p__Firmicutes;c__Bacilli;o__Bacillales;f__Paenibacillaceae
k__Bacteria;p__Firmicutes;c__Bacilli;o__Bacillales;f__Planococcaceae
k__Bacteria;p__Firmicutes;c__Bacilli;o__Bacillales;f__Staphylococcaceae
k__Bacteria;p__Firmicutes;c__Bacilli;o__Gemellales;f__
k__Bacteria;p__Firmicutes;c__Bacilli;o__Gemellales;f__Gemellaceae
k__Bacteria;p__Firmicutes;c__Bacilli;o__Lactobacillales;Other
k__Bacteria;p__Firmicutes;c__Bacilli;o__Lactobacillales;f__
k__Bacteria;p__Firmicutes;c__Bacilli;o__Lactobacillales;f__Aerococcaceae
k__Bacteria;p__Firmicutes;c__Bacilli;o__Lactobacillales;f__Carnobacteriaceae
k__Bacteria;p__Firmicutes;c__Bacilli;o__Lactobacillales;f__Enterococcaceae
k__Bacteria;p__Firmicutes;c__Bacilli;o__Lactobacillales;f__Lactobacillaceae
k__Bacteria;p__Firmicutes;c__Bacilli;o__Lactobacillales;f__Leuconostocaceae
k__Bacteria;p__Firmicutes;c__Bacilli;o__Lactobacillales;f__Streptococcaceae
k__Bacteria;p__Firmicutes;c__Bacilli;o__Turicibacterales;f__Turicibacteraceae
k__Bacteria;p__Firmicutes;c__Clostridia;Other;Other
k__Bacteria;p__Firmicutes;c__Clostridia;o__Clostridiales;Other
k__Bacteria;p__Firmicutes;c__Clostridia;o__Clostridiales;f__
k__Bacteria;p__Firmicutes;c__Clostridia;o__Clostridiales;f__Christensenellaceae
k__Bacteria;p__Firmicutes;c__Clostridia;o__Clostridiales;f__Clostridiaceae
k__Bacteria;p__Firmicutes;c__Clostridia;o__Clostridiales;f__Dehalobacteriaceae
k__Bacteria;p__Firmicutes;c__Clostridia;o__Clostridiales;f__Eubacteriaceae
k__Bacteria;p__Firmicutes;c__Clostridia;o__Clostridiales;f__Lachnospiraceae
k__Bacteria;p__Firmicutes;c__Clostridia;o__Clostridiales;f__Peptococcaceae
k__Bacteria;p__Firmicutes;c__Clostridia;o__Clostridiales;f__Peptostreptococcaceae
k__Bacteria;p__Firmicutes;c__Clostridia;o__Clostridiales;f__Ruminococcaceae
k__Bacteria;p__Firmicutes;c__Clostridia;o__Clostridiales;f__Veillonellaceae
k__Bacteria;p__Firmicutes;c__Clostridia;o__Clostridiales;f__[Mogibacteriaceae]
k__Bacteria;p__Firmicutes;c__Clostridia;o__Clostridiales;f__[Tissierellaceae]
k__Bacteria;p__Firmicutes;c__Erysipelotrichi;o__Erysipelotrichales;f__Erysipelotrichaceae
k__Bacteria;p__Fusobacteria;c__Fusobacteriia;o__Fusobacteriales;Other
k__Bacteria;p__Fusobacteria;c__Fusobacteriia;o__Fusobacteriales;f__Fusobacteriaceae
k__Bacteria;p__Fusobacteria;c__Fusobacteriia;o__Fusobacteriales;f__Leptotrichiaceae
k__Bacteria;p__Gemmatimonadetes;c__Gemmatimonadetes;o__;f__
k__Bacteria;p__Lentisphaerae;c__[Lentisphaeria];o__Victivallales;f__Victivallaceae
k__Bacteria;p__Lentisphaerae;c__[Lentisphaeria];o__Z20;f__R4-45B
k__Bacteria;p__OD1;Other;Other;Other
k__Bacteria;p__Planctomycetes;c__Planctomycetia;Other;Other
k__Bacteria;p__Planctomycetes;c__Planctomycetia;o__Pirellulales;f__Pirellulaceae
k__Bacteria;p__Proteobacteria;Other;Other;Other
k__Bacteria;p__Proteobacteria;c__Alphaproteobacteria;Other;Other
k__Bacteria;p__Proteobacteria;c__Alphaproteobacteria;o__Caulobacterales;f__Caulobacteraceae
k__Bacteria;p__Proteobacteria;c__Alphaproteobacteria;o__RF32;f__
k__Bacteria;p__Proteobacteria;c__Alphaproteobacteria;o__Rhizobiales;Other
k__Bacteria;p__Proteobacteria;c__Alphaproteobacteria;o__Rhizobiales;f__Beijerinckiaceae
k__Bacteria;p__Proteobacteria;c__Alphaproteobacteria;o__Rhizobiales;f__Bradyrhizobiaceae
k__Bacteria;p__Proteobacteria;c__Alphaproteobacteria;o__Rhizobiales;f__Brucellaceae
k__Bacteria;p__Proteobacteria;c__Alphaproteobacteria;o__Rhizobiales;f__Hyphomicrobiaceae
k__Bacteria;p__Proteobacteria;c__Alphaproteobacteria;o__Rhizobiales;f__Methylobacteriaceae
k__Bacteria;p__Proteobacteria;c__Alphaproteobacteria;o__Rhizobiales;f__Phyllobacteriaceae
k__Bacteria;p__Proteobacteria;c__Alphaproteobacteria;o__Rhizobiales;f__Rhizobiaceae
k__Bacteria;p__Proteobacteria;c__Alphaproteobacteria;o__Rhizobiales;f__Xanthobacteraceae
k__Bacteria;p__Proteobacteria;c__Alphaproteobacteria;o__Rhodobacterales;f__Rhodobacteraceae
k__Bacteria;p__Proteobacteria;c__Alphaproteobacteria;o__Rhodospirillales;f__Acetobacteraceae
k__Bacteria;p__Proteobacteria;c__Alphaproteobacteria;o__Rhodospirillales;f__Rhodospirillaceae
k__Bacteria;p__Proteobacteria;c__Alphaproteobacteria;o__Rickettsiales;f__
k__Bacteria;p__Proteobacteria;c__Alphaproteobacteria;o__Rickettsiales;f__mitochondria
k__Bacteria;p__Proteobacteria;c__Alphaproteobacteria;o__Sphingomonadales;Other
k__Bacteria;p__Proteobacteria;c__Alphaproteobacteria;o__Sphingomonadales;f__
k__Bacteria;p__Proteobacteria;c__Alphaproteobacteria;o__Sphingomonadales;f__Erythrobacteraceae
k__Bacteria;p__Proteobacteria;c__Alphaproteobacteria;o__Sphingomonadales;f__Sphingomonadaceae
k__Bacteria;p__Proteobacteria;c__Betaproteobacteria;Other;Other
k__Bacteria;p__Proteobacteria;c__Betaproteobacteria;o__ASSO-13;f__
k__Bacteria;p__Proteobacteria;c__Betaproteobacteria;o__Burkholderiales;Other
k__Bacteria;p__Proteobacteria;c__Betaproteobacteria;o__Burkholderiales;f__Alcaligenaceae
k__Bacteria;p__Proteobacteria;c__Betaproteobacteria;o__Burkholderiales;f__Comamonadaceae
k__Bacteria;p__Proteobacteria;c__Betaproteobacteria;o__Neisseriales;f__Neisseriaceae
k__Bacteria;p__Proteobacteria;c__Betaproteobacteria;o__SC-I-84;f__
k__Bacteria;p__Proteobacteria;c__Deltaproteobacteria;Other;Other
k__Bacteria;p__Proteobacteria;c__Deltaproteobacteria;o__Desulfovibrionales;f__Desulfovibrionaceae
k__Bacteria;p__Proteobacteria;c__Epsilonproteobacteria;o__Campylobacterales;f__Campylobacteraceae
k__Bacteria;p__Proteobacteria;c__Gammaproteobacteria;Other;Other
k__Bacteria;p__Proteobacteria;c__Gammaproteobacteria;o__Aeromonadales;f__Succinivibrionaceae
k__Bacteria;p__Proteobacteria;c__Gammaproteobacteria;o__Alteromonadales;Other
k__Bacteria;p__Proteobacteria;c__Gammaproteobacteria;o__Alteromonadales;f__Alteromonadaceae
k__Bacteria;p__Proteobacteria;c__Gammaproteobacteria;o__Alteromonadales;f__Idiomarinaceae
k__Bacteria;p__Proteobacteria;c__Gammaproteobacteria;o__Alteromonadales;f__[Chromatiaceae]
k__Bacteria;p__Proteobacteria;c__Gammaproteobacteria;o__Enterobacteriales;f__Enterobacteriaceae
k__Bacteria;p__Proteobacteria;c__Gammaproteobacteria;o__Legionellales;f__
k__Bacteria;p__Proteobacteria;c__Gammaproteobacteria;o__Oceanospirillales;f__Alcanivoracaceae
k__Bacteria;p__Proteobacteria;c__Gammaproteobacteria;o__Oceanospirillales;f__Halomonadaceae
k__Bacteria;p__Proteobacteria;c__Gammaproteobacteria;o__Pasteurellales;f__Pasteurellaceae
k__Bacteria;p__Proteobacteria;c__Gammaproteobacteria;o__Pseudomonadales;Other
k__Bacteria;p__Proteobacteria;c__Gammaproteobacteria;o__Pseudomonadales;f__Moraxellaceae
k__Bacteria;p__Proteobacteria;c__Gammaproteobacteria;o__Pseudomonadales;f__Pseudomonadaceae
k__Bacteria;p__Proteobacteria;c__Gammaproteobacteria;o__Xanthomonadales;f__Sinobacteraceae
k__Bacteria;p__Proteobacteria;c__Gammaproteobacteria;o__Xanthomonadales;f__Xanthomonadaceae
k__Bacteria;p__Spirochaetes;c__Spirochaetes;o__M2PT2-76;f__
k__Bacteria;p__Spirochaetes;c__Spirochaetes;o__Sphaerochaetales;f__Sphaerochaetaceae
k__Bacteria;p__Spirochaetes;c__Spirochaetes;o__Spirochaetales;f__Spirochaetaceae
k__Bacteria;p__Synergistetes;c__Synergistia;o__Synergistales;f__
k__Bacteria;p__TM7;c__TM7-1;o__;f__
k__Bacteria;p__TM7;c__TM7-3;Other;Other
k__Bacteria;p__TM7;c__TM7-3;o__CW040;f__F16
k__Bacteria;p__TM7;c__TM7-3;o__EW055;f__
k__Bacteria;p__Tenericutes;Other;Other;Other
k__Bacteria;p__Tenericutes;c__Mollicutes;o__Acholeplasmatales;f__Acholeplasmataceae
k__Bacteria;p__Tenericutes;c__Mollicutes;o__Anaeroplasmatales;f__Anaeroplasmataceae
k__Bacteria;p__Tenericutes;c__Mollicutes;o__Mycoplasmatales;f__Mycoplasmataceae
k__Bacteria;p__Tenericutes;c__Mollicutes;o__RF39;f__
k__Bacteria;p__Tenericutes;c__RF3;o__ML615J-28;f__
k__Bacteria;p__Verrucomicrobia;c__Opitutae;o__HA64;f__
k__Bacteria;p__Verrucomicrobia;c__Opitutae;o__[Cerasicoccales];f__[Cerasicoccaceae]
k__Bacteria;p__Verrucomicrobia;c__Verruco-5;o__WCHB1-41;f__RFP12
k__Bacteria;p__Verrucomicrobia;c__Verrucomicrobiae;o__Verrucomicrobiales;f__Verrucomicrobiaceae
k__Bacteria;p__[Thermi];c__Deinococci;o__Deinococcales;f__Deinococcaceae
