# Supplementary figures and images for: Delayed differentiation of vaginal and uterine microbiomes in dairy cows developing postpartum endometritis
Source: PLoS One. 2019 Jan 10;14(1):e0200974. doi: 10.1371/journal.pone.0200974 (PMC6328119; doi:10.1371/journal.pone.0200974)

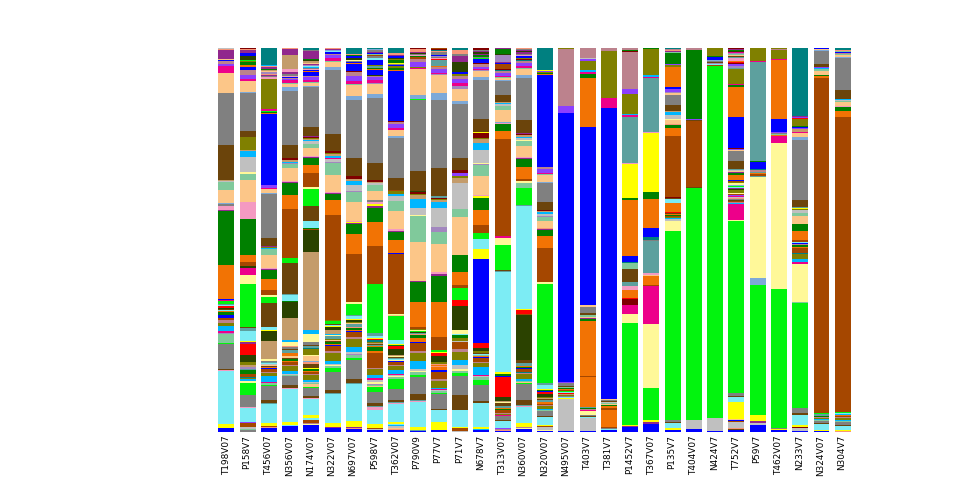

Supplement: S4 Fig — Original output generated by QIIME. To visualise it double click on bar_charts.html. (ZIP) [file pone.0200974.s006.zip › Figure S4/charts/2nBJQemMr0No7WWz4dXjpIsnDrOYw4.png]

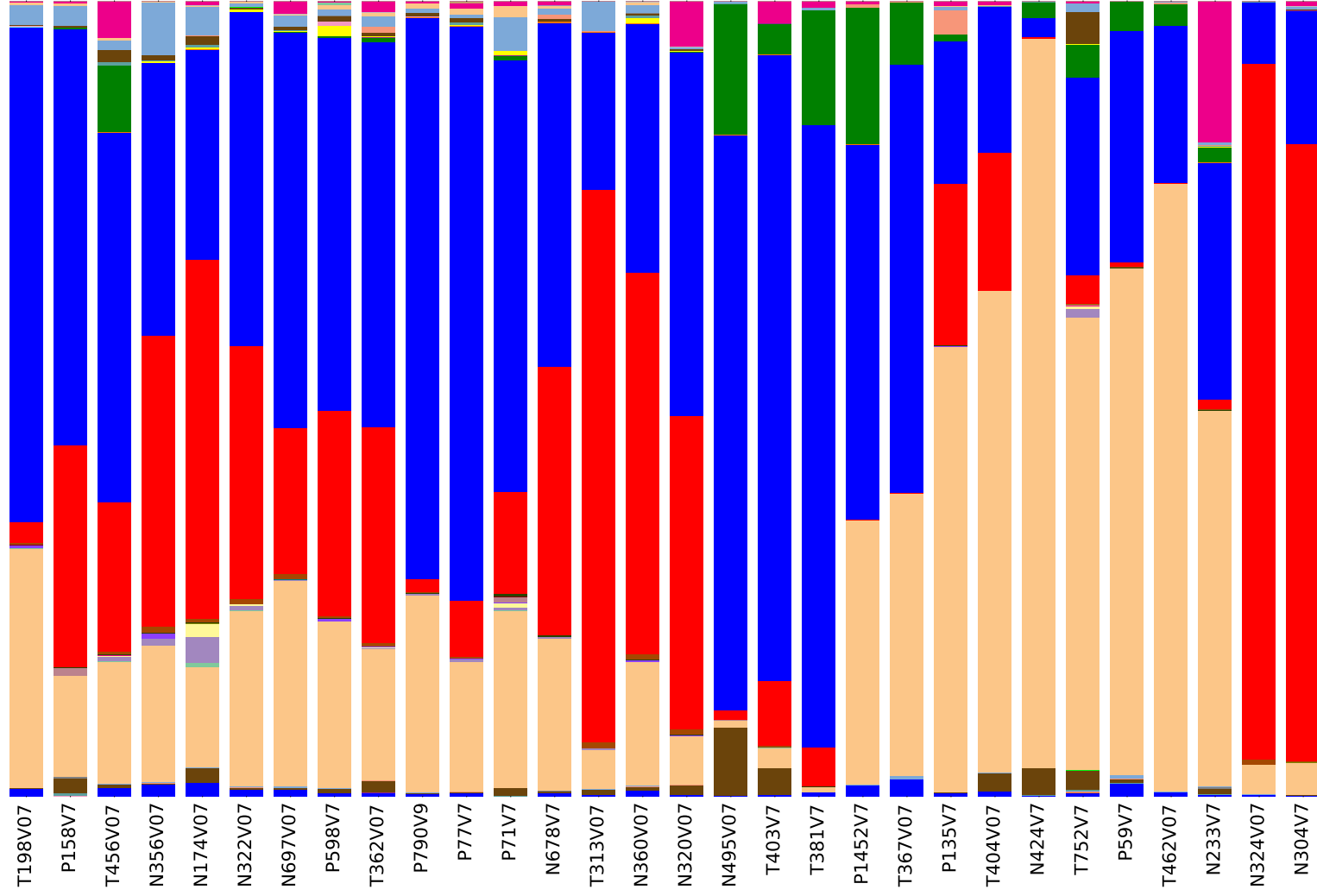

Supplement: S4 Fig — Original output generated by QIIME. To visualise it double click on bar_charts.html. (ZIP) [file pone.0200974.s006.zip › Figure S4/charts/7Cp0a0DCkUfCaHr5nhsc9amtuxQUK0.pdf]

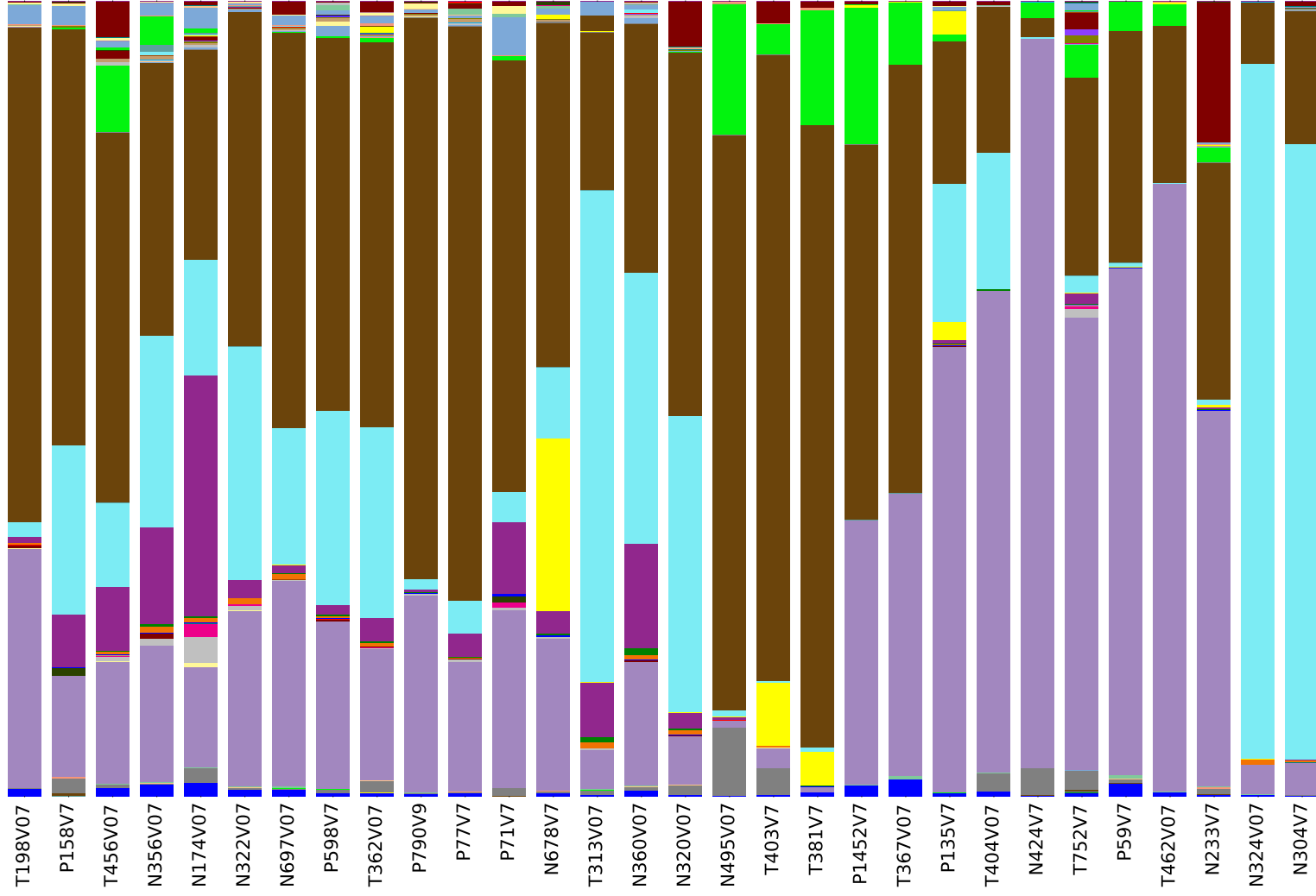

Supplement: S4 Fig — Original output generated by QIIME. To visualise it double click on bar_charts.html. (ZIP) [file pone.0200974.s006.zip › Figure S4/charts/C66choOx1XzpxwHfglzXW481U3Rtay.pdf]

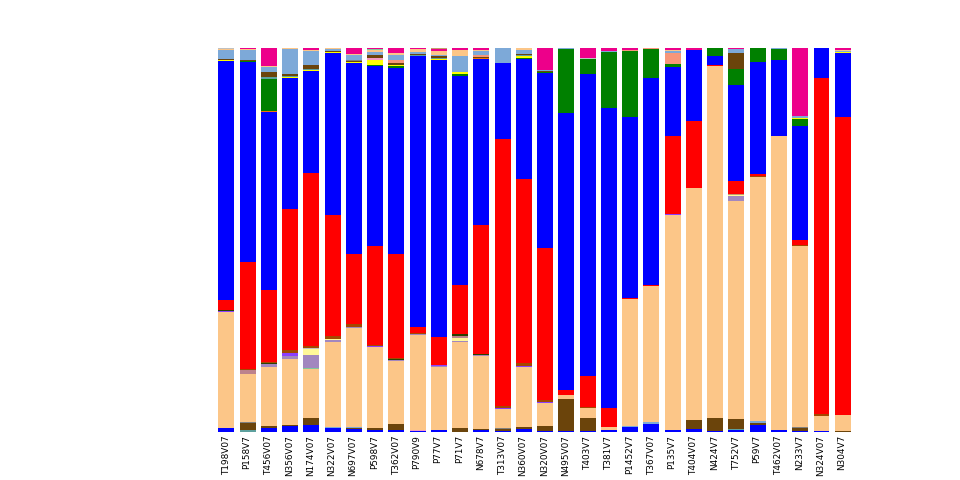

Supplement: S4 Fig — Original output generated by QIIME. To visualise it double click on bar_charts.html. (ZIP) [file pone.0200974.s006.zip › Figure S4/charts/eBS9C7dWWZM30zwPdT8QOjW40KfYD9.png]

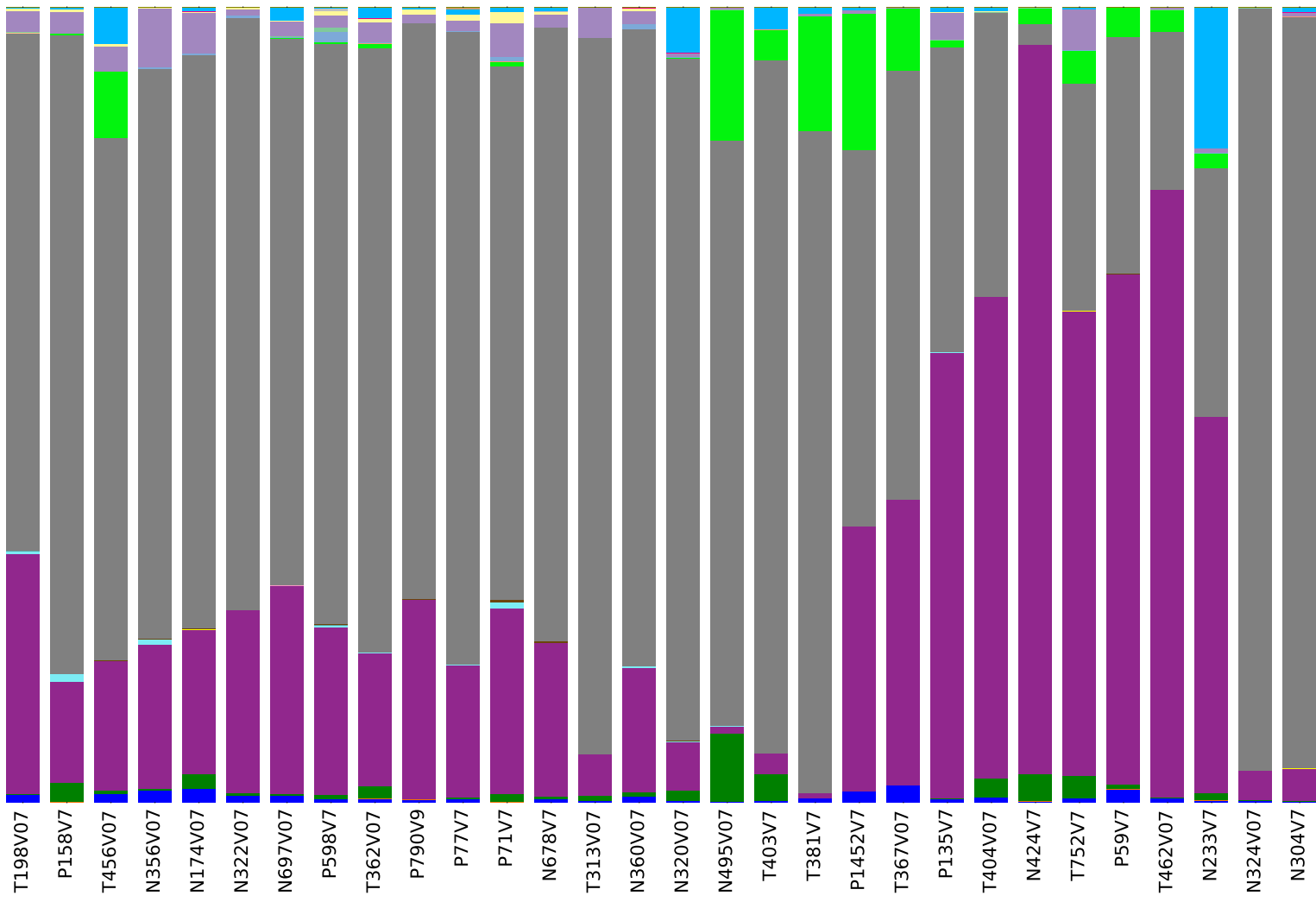

Supplement: S4 Fig — Original output generated by QIIME. To visualise it double click on bar_charts.html. (ZIP) [file pone.0200974.s006.zip › Figure S4/charts/M5LGrztBRWZHHNGpsXEMgzzxLZAisz.pdf]

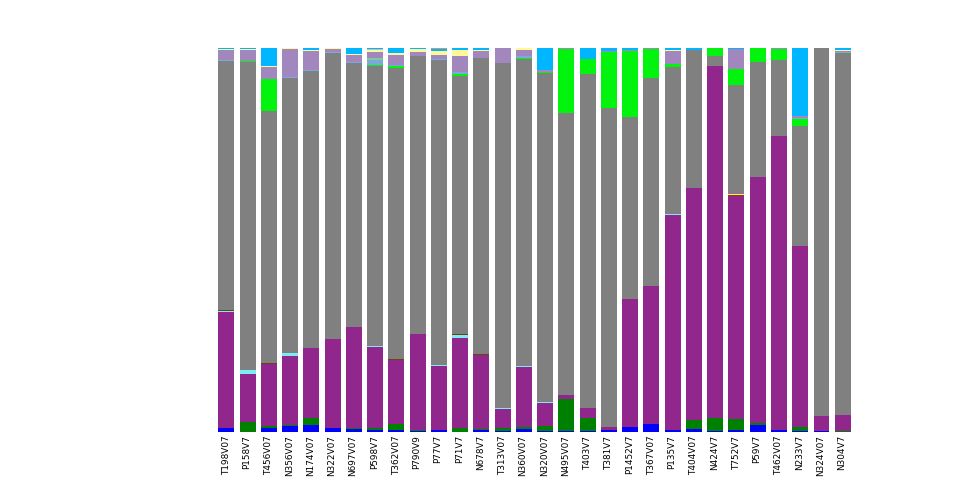

Supplement: S4 Fig — Original output generated by QIIME. To visualise it double click on bar_charts.html. (ZIP) [file pone.0200974.s006.zip › Figure S4/charts/MtCoXE44sIKHPIy1aZDYncrCSc2azG.png]

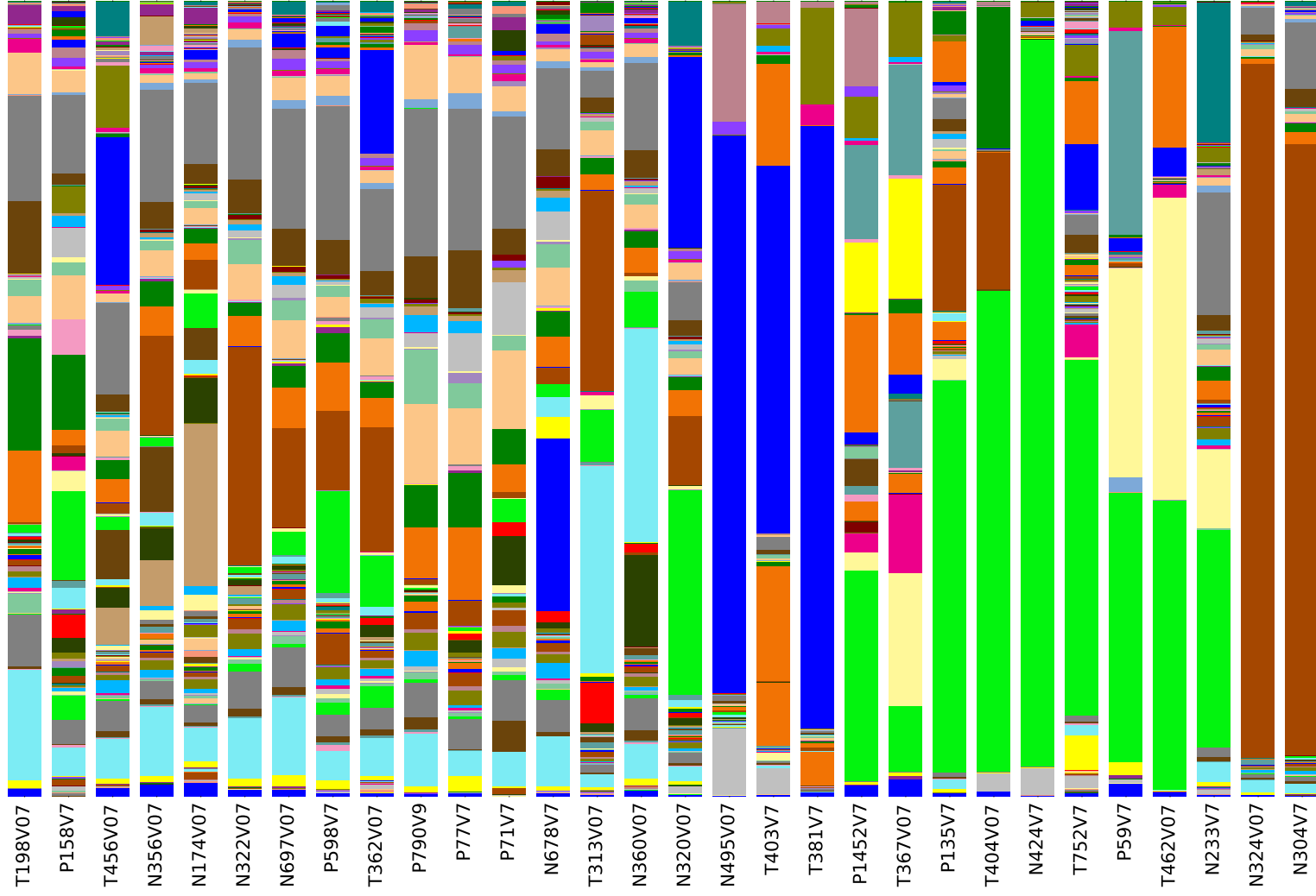

Supplement: S4 Fig — Original output generated by QIIME. To visualise it double click on bar_charts.html. (ZIP) [file pone.0200974.s006.zip › Figure S4/charts/muATzXRTjBss30T2dLzR6hJsYCHcoR.pdf]

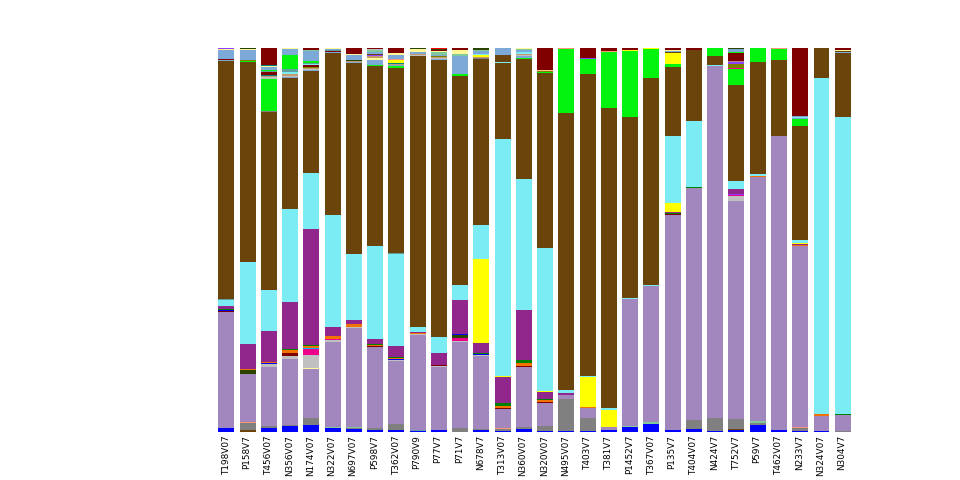

Supplement: S4 Fig — Original output generated by QIIME. To visualise it double click on bar_charts.html. (ZIP) [file pone.0200974.s006.zip › Figure S4/charts/sZ7JijCfSoGszo0E0RuyzXLmljCn0t.png]

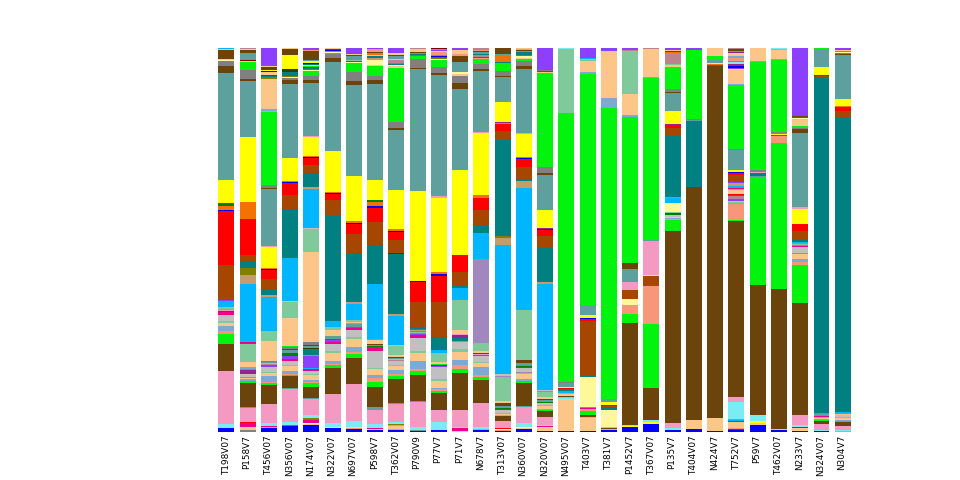

Supplement: S4 Fig — Original output generated by QIIME. To visualise it double click on bar_charts.html. (ZIP) [file pone.0200974.s006.zip › Figure S4/charts/TXgz38RRizZhyyZ1fpeSyCdk3gq0jj.png]

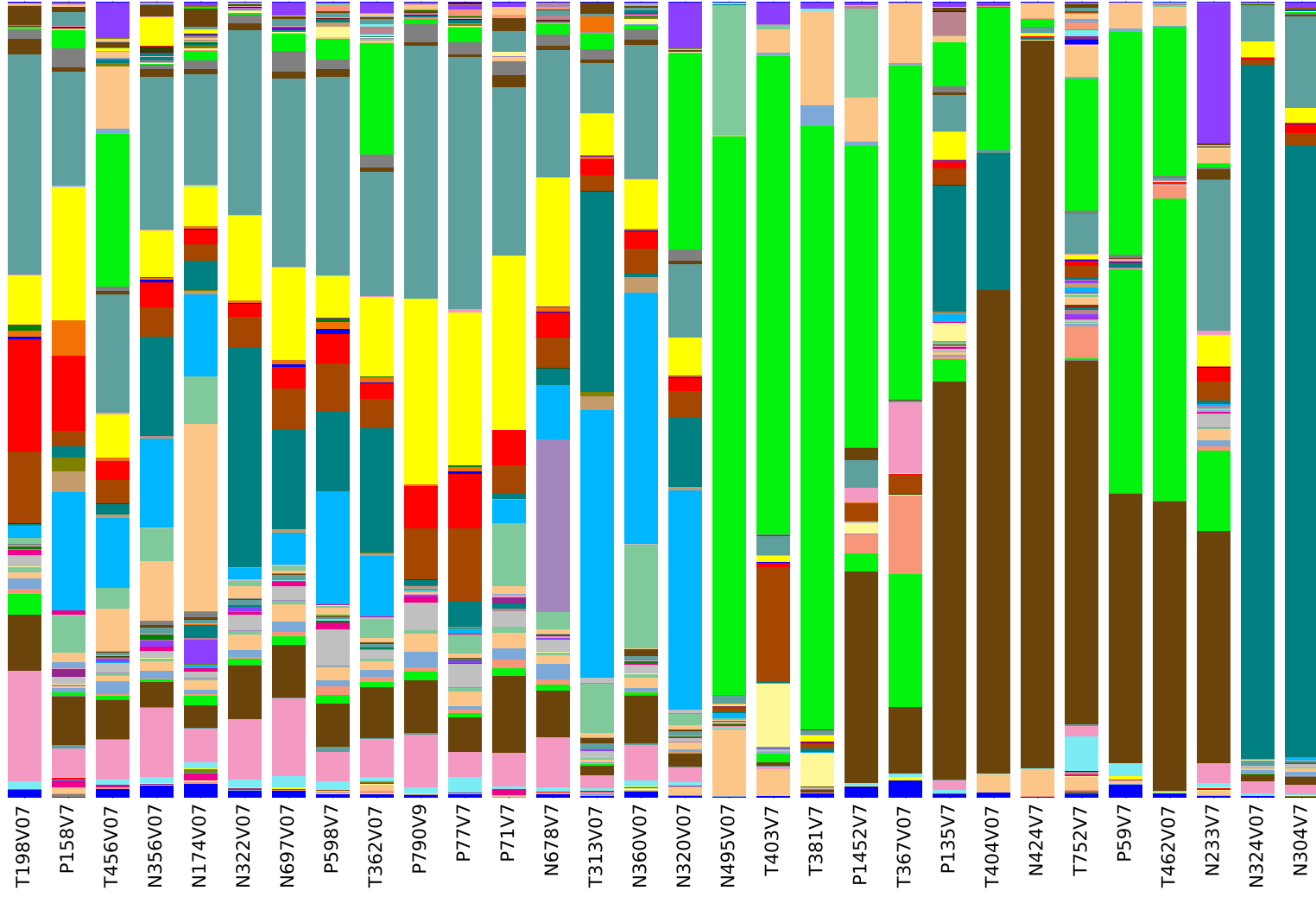

Supplement: S4 Fig — Original output generated by QIIME. To visualise it double click on bar_charts.html. (ZIP) [file pone.0200974.s006.zip › Figure S4/charts/Y7WA9phMFA6PmW651bxtkw7frzpMmh.pdf]
